# COLMARvista: an open source 2D and pseudo-3D NMR spectral processing, visualization, and analysis software in JavaScript

**DOI:** 10.1007/s10858-025-00465-y

**Published:** 2025-03-28

**Authors:** Dawei Li, Rafael Brüschweiler

**Affiliations:** 1 Department of Chemistry and Biochemistry, The Ohio State University, Columbus, OH 43210 USA; 2 Department of Biological Chemistry and Pharmacology, The Ohio State University, Columbus, OH 43210 USA

**Keywords:** NMR software, NMR spectral visualization, JavaScript, Open-source software

## Abstract

COLMARvista is presented as a new, highly versatile software for the easy and intuitive processing and visual inspection of 2D and pseudo-3D NMR data both for uniformly and non-uniformly sampled datasets. COLMARvista allows fully autonomous processing of spectra, including zero-filling, apodization, water suppression, Fourier transformation, and phase correction. Its full integration with DEEP Picker and Voigt Fitter programs allows the automated deconvolution and reconstruction of the experimental spectra for highly quantitative analysis, from compound concentration determination to the extraction of cross-peak specific relaxation parameters, even for signals affected by significant overlap with other peaks. COLMARvista is based on JavaScript and, hence, it is computer-architecture and operating-system independent including its advanced graphics. It runs on all recent web browsers and does not require a potentially elaborate operating-system dependent installation. COLMARvista may serve as a paradigm also for other software projects to prevent the stockpiling of once powerful legacy software that became frozen in time, thereby ensuring continuing progress of the NMR field and its software for future generations of NMR spectroscopists.

## Introduction

Over time, nuclear Magnetic Resonance (NMR) spectroscopy has steadily evolved into a powerful tool that is now widely used in many different fields, such as chemistry, biology, biomedicine, and materials science. It provides detailed information about the molecular structure, dynamics, and interactions of molecules and materials by measuring the response of atomic nuclear spins to external magnetic fields. NMR is essential, for example, for identifying unknown substances, determining the atomic structure and dynamics of molecules and materials, characterizing protein–ligand interactions, or monitoring chemical reactions. Its intrinsic non-destructive nature and high resolution make it an indispensable mainstay in research and quality control across diverse fields. While NMR spectroscopy provides unparalleled insights, much of the raw information is indirect, making the extraction of underlying atomistic details about their structure, dynamics and interactions quite complex and demanding. Since the early days, computer-assisted processing and analysis have played a key role in NMR applications. Well-designed software tools help streamline complex workflows, enhance accessibility to a broad range of users, assist in the improved reproducibility of research results, and assure the software’s maintainability and longevity.

The past several decades have seen a growing number of NMR software to record, visualize, and analyze NMR data. This includes both commercial software, such as Topspin and MNova, and open-source software, such as NMRPipe (Delaglio et al. 1995), CCPN (Vranken et al. 2005), Sparky (Lee et al. 2015; Goddard and Kneller 2008), NMRFx (Johnson 2018), POKY (Lee et al. 2021), ssNake (Meerten et al. 2019), nmrium (Patiny et al. 2024) and nmrGlue (Helmus and Jaroniec 2013) among many others (Blanton 2003; Beek 2007; Nowling et al. 2011; Short et al. 2011; Lewis et al. 2019; Gunther et al. 2000). Open-source NMR software is often maintained by dedicated volunteers, often graduate students, postdocs, and staff scientists, contributing their time and expertise to support the wider community. However, this model faces significant challenges in keeping up with the rapid evolution of NMR-specific computational tools, operating systems (OS), and software libraries, including computer graphics. Frequent updates to operating system platforms and dependencies demand continuous maintenance and adaptation, which can be highly resource intensive. Consequently, open-source software programs often struggle with compatibility issues and performance gaps as the software ages. An increasing number of NMR users rely on cloud platforms like NMRBox (Maciejewski et al. 2017), which provides access to "legacy" software no longer supported on current computational platforms and potentially introducing other dependencies beyond the user’s control. Although legacy software has the benefit of being tried and tested, it forces NMR users to work with outdated software frozen in the past, which invariably slows progress of the NMR field as a whole.

To overcome these challenges, we introduce a new standalone NMR software named COLMARvista, which emerged from our COLMAR web-server suite. It allows both autonomous and supervised 2D and pseudo-3D NMR spectrum processing and analysis, including spectral visualization, peak picking and peak fitting. COLMARvista is built on a web platform, i.e. it runs entirely within a browser window on a user’s computer without transferring or sharing any data with a remote server. This design provides several advantages: (1) COLMARvista, including its sophisticated graphics tools, is universally compatible with all major operating systems, as long as they support a modern web browser; (2) it does not require any OS-specific installation and can be accessed in the cloud or downloaded as an HTML file package for local use; and (3) it is based on JavaScript, a primary programming language for web applications, which prioritizes backward compatibility by adhering to the principle of "don't break the web," ensuring that code written today will still function on future browsers, e.g., 10 or more years from now. In addition, COLMARvista is open source, interactive, welcomes input and contributions by the community, and it emphasizes efficiency by leveraging the latest web technologies, such as WebGL (WebGPU 2025) and WebAssembly (2019).

## Software design

Cross-platform Graphic User Interface programming is complex as it involves creating user interfaces that work seamlessly across different operating systems, each with its own unique set of features, design conventions, and APIs, such as "Windows Forms" on Windows, "Cocoa" on macOS, or "GTK + " on Linux. While cross-platform frameworks, such as Qt, aim to provide a unified approach, they often require workarounds and adjustments to account for platform-specific differences, such as file systems, graphics rendering, or event handling. This often significantly adds to development efforts. To address this problem, COLMARvista, including its GUI, was developed using web technologies that provide a unified and consistent user experience on all major browsers, such as Google Chrome, Apple Safari, MS Edge and Mozilla Firefox, independent of the underlying OS. This also bypasses the often-tricky software installation step, including the installation of all necessary libraries.

Besides the typical GUI elements required for any computer program with user input, such as boxes, text fields, buttons, and input areas, the web platform also provides essential tools for the visualization of 2D NMR spectra. In COLMARvista, all spectral plotting, including axes, labels, and symbols for peaks, is handled by HTML Scalable Vector Graphics (SVG) elements, which are highly flexible for drawing all kinds of graphs. For the visualization of 2D NMR spectra, contour plots remain the most widely used means due to their intuitive and yet quantitative nature. A complex NMR spectrum, such as a TOCSY spectrum, may contain tens of thousands of line segments in its contours, making zooming and panning operations computationally demanding, thereby causing significant slow-down. To address this issue, COLMARvista uses a WebGL 2025 based technique to handle contour plotting, effectively shifting the computational burden from the CPU to the GPU. This approach enables instant contour refreshing when users zoom in or out or pan the spectral plot. COLMARvista also supports alternative and customizable ways of visualizing 2D NMR spectra other than the traditional contour plots, such 3D terrace plots and 3D surface plots (vide infra).

In addition to spectral visualization, COLMARvista provides a standard NMR data processing pipeline that converts time-domain data into frequency-domain data. This is achieved through apodization, Fast Fourier Transformation (FFT), and phase correction. On the spectral analysis side, COLMARvista is fully integrated with the DEEP Picker and Voigt Fitter programs to deconvolute spectra into individual peaks in a highly quantitative manner (Li et al. 2021; Li et al. 2022). These programs, already available in our DEEP Picker package (Li et al. 2021; Li n.d.), have been ported to the web environment using WebAssembly (WebAssembly 2019), which requires minimal source code modifications and retains approximately 80–90% of the execution speed of OS-specific native executables.

The web-centric design of COLMARvista is not without limitations. Since it is a web application, it cannot access the local file system directly and all input, intermediate, and output files generated or used by COLMARvista must be stored in a virtual file system specified in the browser. This virtual file system currently has a hard size limit of 4 GB, which however is expected to be increased in future browser releases. Fortunately, for most 2D and pseudo-3D NMR datasets, this storage capacity is sufficient.

## Key functionalities of COLMARvista

COLMARvista can read processed frequency-domain data in several different file formats, including NMRPipe (Delaglio et al. 1995) (with its file extension ft2), UCSF Sparky (Lee et al. 2015; Goddard and Kneller 2008) (file extension ucsf), and Topspin txt files (generated in Topspin using the "totxt" command). One of the major advantages of COLMARvista is its WebGL-based contour plot visualization. For a typical 2D HSQC spectrum the initial calculation of contours from spectral data may take from several seconds, such as a HSQC, to up to a minute. For more complex spectra with a large number of cross-peaks, such as a TOCSY or NOESY spectrum. However, common subsequent operations, such as zooming and panning, are instantaneous even when working with multiple overlaid contour plots owing to the high computational efficiency of modern GPU’s. In addition to the main 2D plot, COLMARvista provides two 1D spectral windows, which can display either projections or selected 1D cross-sections based on user preference. The 2D plot supports intuitive zooming using a brush tool, allowing users to select specific regions for more detailed inspection. Meanwhile, the 1D plots feature zooming via the mouse wheel (or equivalent) and panning through mouse dragging. Both 1D plots are fully synchronized with the main 2D plot, i.e. zooming or panning in one plot automatically updates the others, thereby maintaining consistent views across all windows. More detailed information is available in the software manual.

**Fig. 1:**
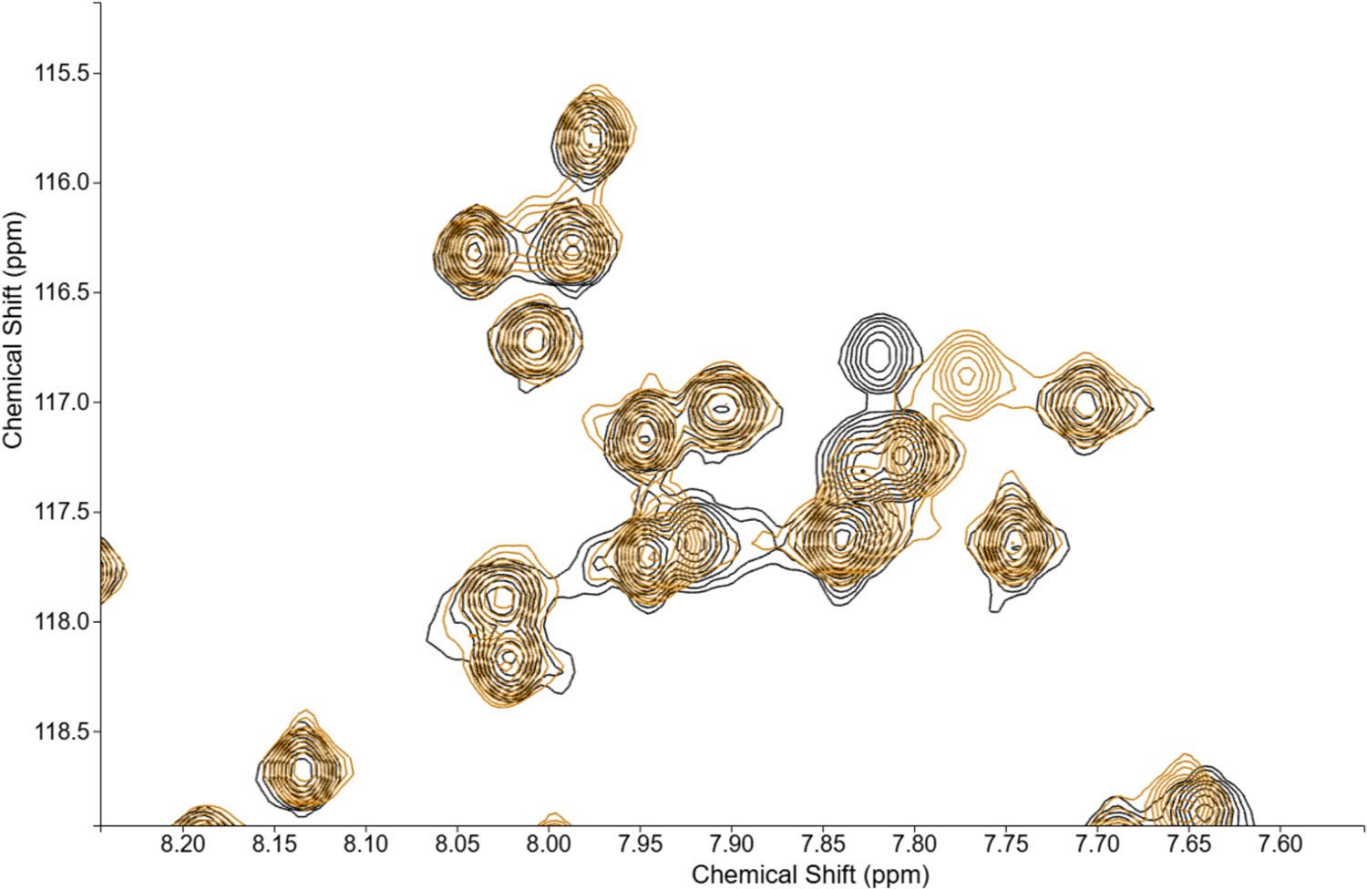
Visualization of the superposition of a selected region of two 2D ^1^H-^15^N HSQC spectra of two different samples of K-Ras (in black and gold) by COLMARvista, highlighting chemical shift perturbations (measured at 850 MHz proton frequency)

**Fig. 2:**
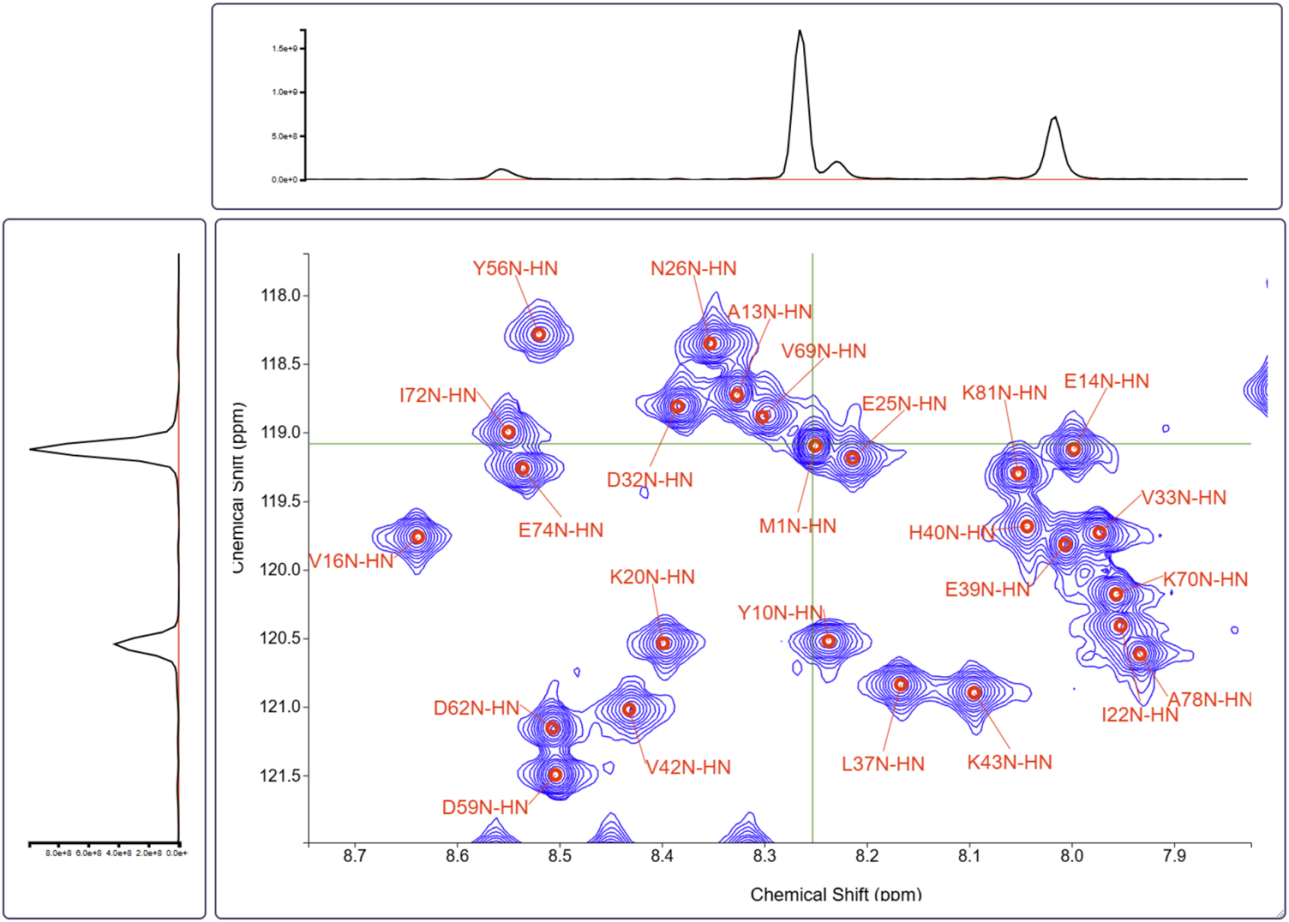
A 2D ^1^H-^15^N HSQC spectrum of colicin E7 immunity protein Im7 with selected peaks and their assignments (indicated by red labels). 1D cross-sections along the green lines in the 2D spectrum are displayed on the left and top of the contour plot

Users can overlay an essentially unlimited number of spectra in COLMARvista, each with its own chemical shift reference. This high level of versatility allows for comprehensive comparative analysis across many spectral datasets as is often required in practice. Users can also rearrange the order of overlaid spectra by simply dragging and dropping them, ensuring that the most relevant spectrum is displayed prominently in the foreground. Additionally, the color of each contour plot can be fully customized according to the user’s preferences, making it easy to visually distinguish the different spectra. To further enhance the visualization experience, COLMARvista includes options for adjusting contour levels. The software also supports the export of images for blogs and presentations. Figure 1 shows an example of two superimposed solution NMR protein ^1^H-^15^N HSQC spectra, highlighting chemical shift perturbations. Additionally, COLMARvista supports the display of peak labels, such as assignments, next to each peak. The label positions are automatically arranged for optimal visualization and minimal mutual overlap and, if desired, allowing easy manual adjustment as illustrated in Fig. 2.

**Fig. 3:**
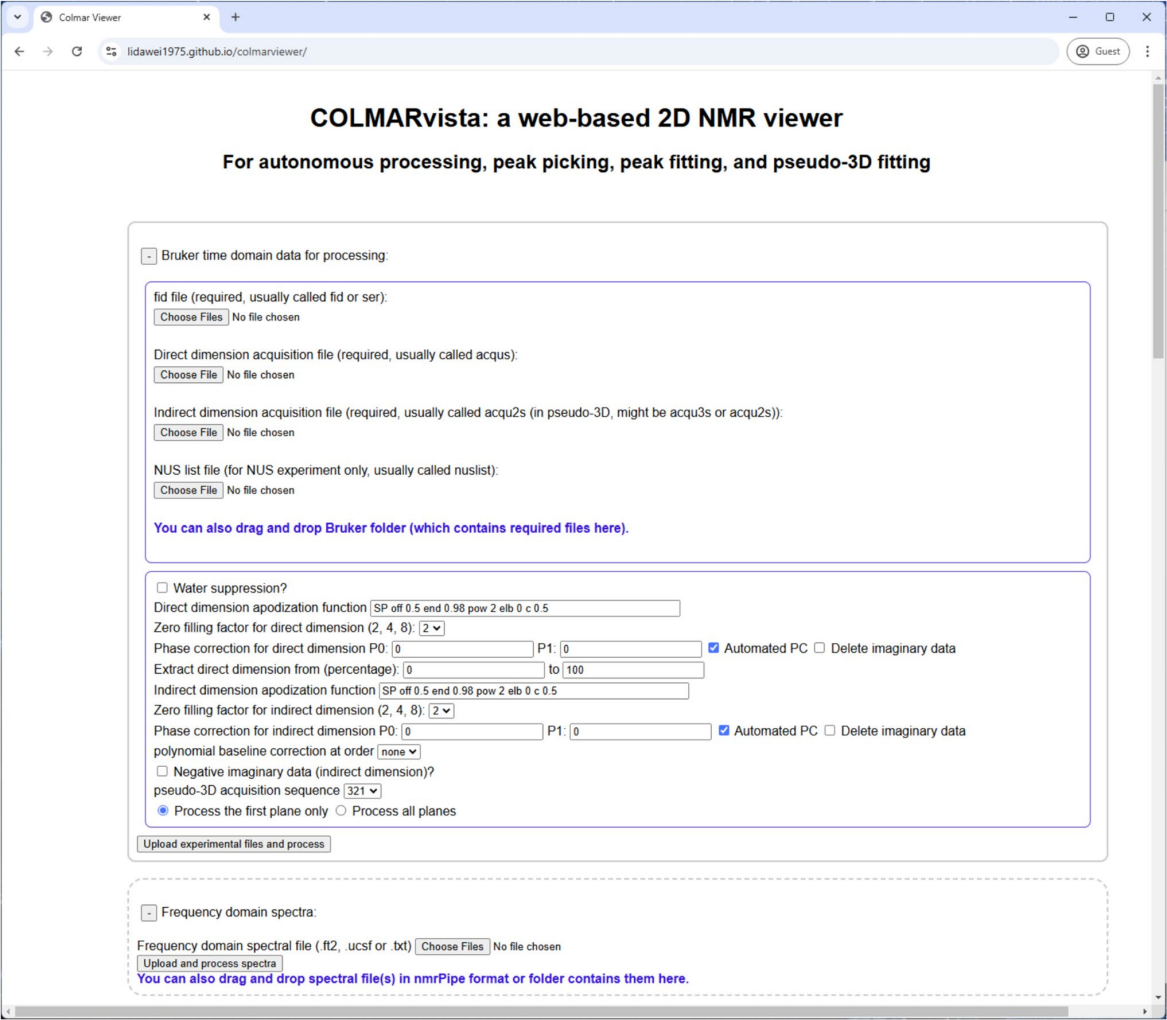
Screenshot of the COLMARvista web application. COLMARvista accepts both pre-processed 2D NMR spectral files in the frequency domain or, alternatively, it can directly process Bruker time-domain data (Topspin 3.0 and later versions). Its processing options, such as apodization, zero-filling, phase correction and extraction, closely mirror those available in NMRPipe and include an automatic phase correction tool

**Fig. 4:**
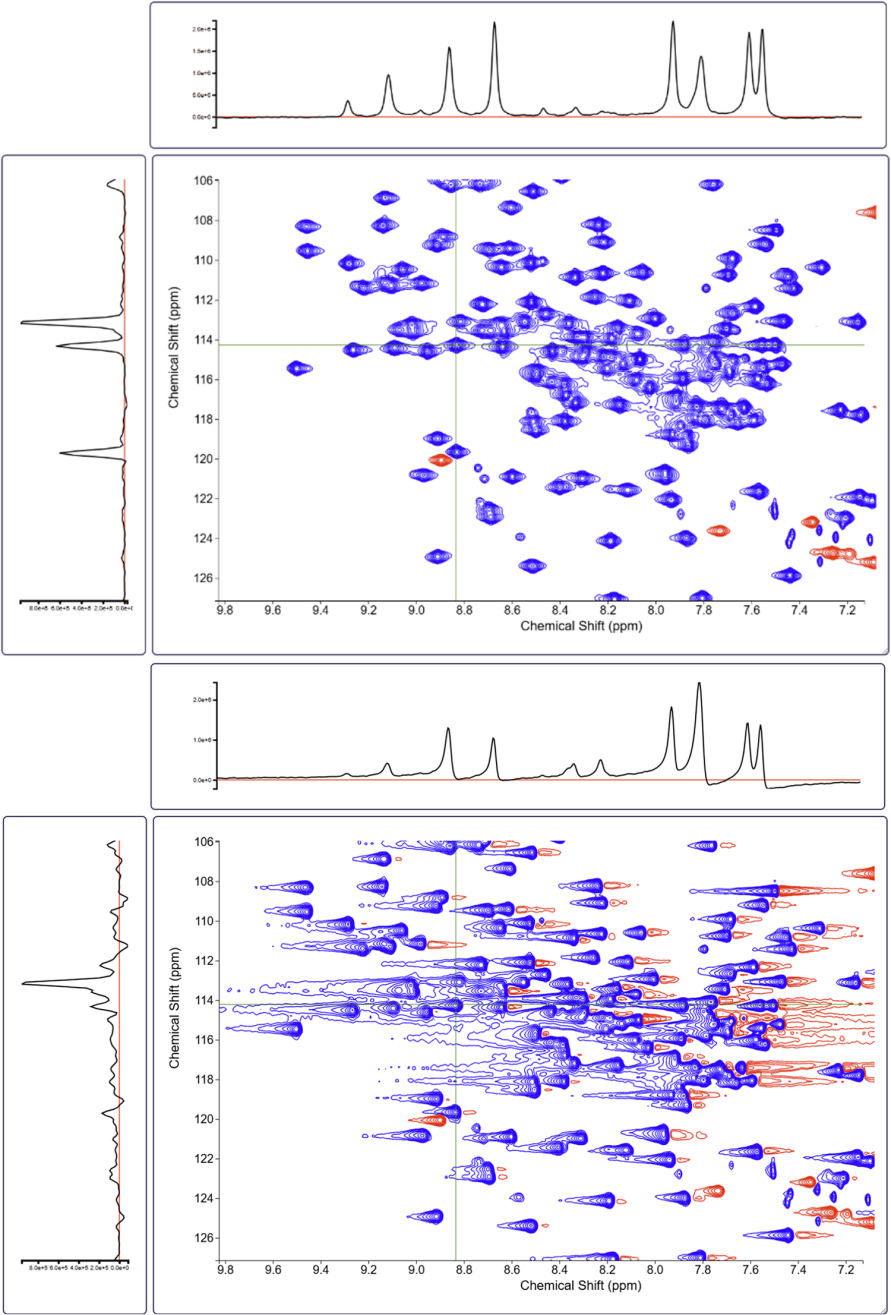
Selected region of a protein 2D ^1^H-^15^N HSQC spectrum of K-Ras **a** with autonomous 0th and 1st order phase correction and **b** without phase correction along the direct ^1^H dimension (phase correction along the indirect ^15^N dimension was applied in both spectra using the phase information defined by the pulse sequence). The cross-section along the direct dimension in (**b**) displays clear phase errors, whereas (**a**) is free from phase errors

In addition to visualization, COLMARvista offers essential tools for the processing of 2D or pseudo-3D time-domain data in Bruker format (version 3.0 or later required). Once users upload the time-domain data (i.e. free induction decays (FIDs)), COLMARvista reads the data and performs a series of predefined processing steps. These include Fast Fourier Transformation (FFT), apodization using user-specified window functions, zero-filling according to user-defined number of folds, and automatic phase correction. If users are not satisfied with the automatic phase correction, COLMARvista provides easy-to-use tools for additional manual phase adjustments to ensure optimal results. For experiments with substantial water signals, COLMARvista also includes automated water solvent suppression by removing the zero-frequency signal from the FID prior to FFT. The overall workflow design, including the syntax of window functions, closely mirrors that of the NMRPipe software, making it intuitive for users familiar with existing NMR data processing software. A screenshot of COLMARvista’s processing interface is shown in Fig. 3 with the fields users can fill in, if the default values need to be changed. Figure 4 depicts the same selected region of a 2D ^1^H-^15^N HSQC protein spectrum (a) with automatic phase correction and (b) without phase correction along the direct dimension. Furthermore, 1D cross-sections along both dimensions are shown in the figures.

In addition to directly processing Bruker-format time-domain data, COLMARvista can also serve as a spectrum visualization and manual phase correction tool as an alternative to NMRDraw to extract phasing information for spectral processing with NMRPipe. Specifically, users can first process time-domain data in NMRPipe, save the frequency-domain spectrum with the imaginary components retained, and then load it into COLMARvista. The phase correction values obtained with COLMARvista, either fully automated or manually, can then be seamlessly integrated into the NMRPipe processing script, following a workflow analogous to that of NMRPipe/NMRDraw.

COLMARvista also allows processing of non-uniformly sampled (NUS) 2D experiments using the SMILE algorithm (Ying et al. 2017). To process NUS data, users simply upload a "nuslists" file alongside the corresponding spectral data files, which automatically designates it as an NUS dataset in COLMARvista. For NUS experiments, users must provide phase correction parameters along the indirect dimension, which are known in most cases directly from the pulse sequence used (see also Fig. 4). COLMARvista will then automatically handle the processing along the direct dimension, including phase correction. Next, the time-domain is fully, i.e. uniformly, reconstructed along the indirect dimension using the SMILE algorithm, which is followed by standard processing along the indirect dimension. As with uniformly sampled experiments, users can then customize within COLMARvista phase correction parameters, apply user-defined window functions, and specify the amount of zero-filling to be used.

**Fig. 5:**
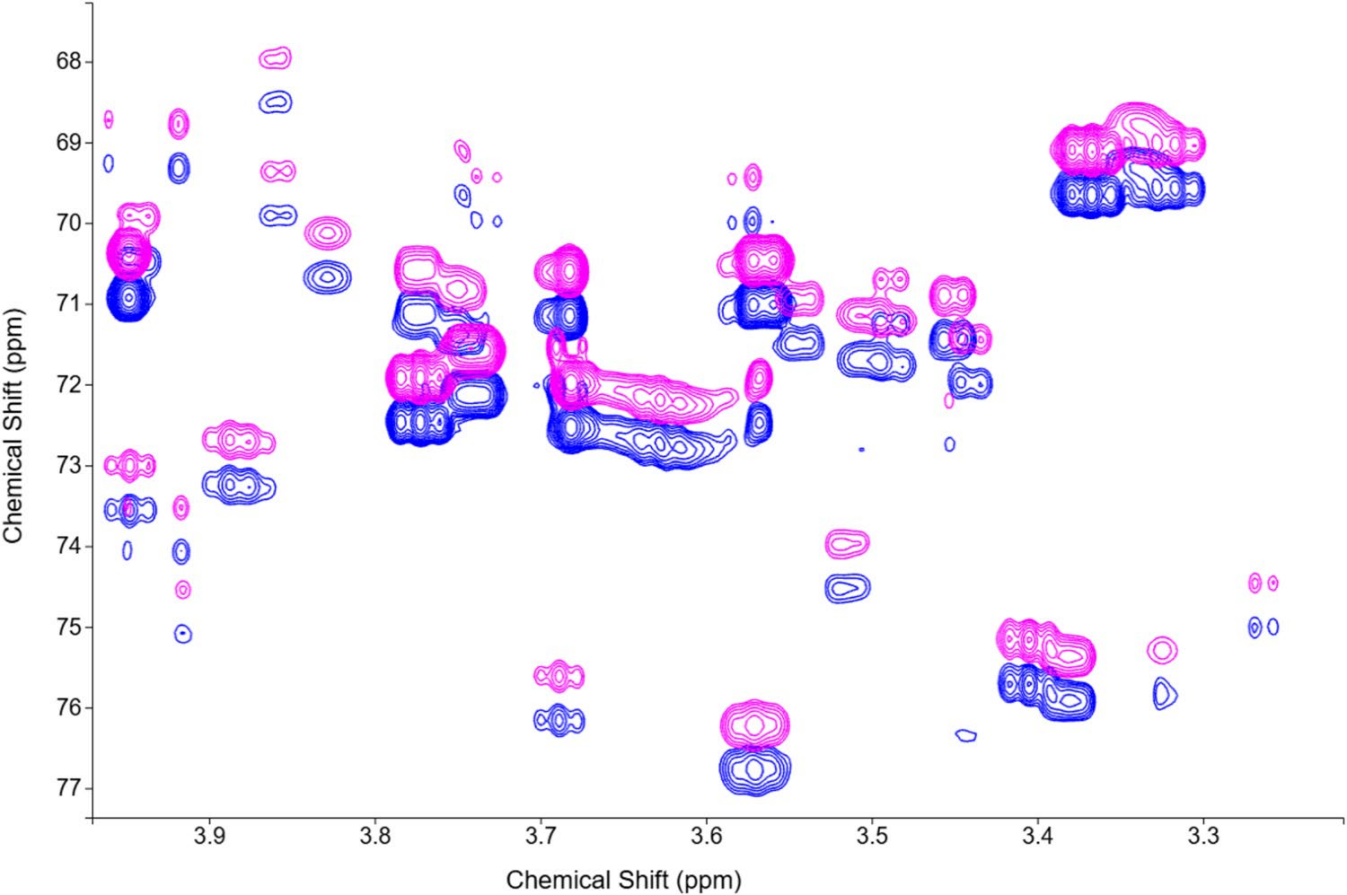
A selected region of a 2D ^1^H-^13^C HSQC spectrum of a biofilm metabolomics sample of P. aeruginosa (blue) together with the reconstructed spectrum (magenta) from peak deconvolution using the DEEP Picker and Voigt Fitter programs built in COLMARvista. The reconstructed spectrum was shifted by a uniform frequency offset along the indirect ^13^C dimension for better visualization

Furthermore, COLMARvista serves as a convenient graphical user interface (GUI) for our DEEP Picker (Li et al. 2021) and Voigt Fitter programs (Li et al. 2022), which deconvolute 2D NMR spectra into individual peaks with high accuracy, even for spectra with strong peak overlaps. The spectral deconvolution step plays a critical role for most NMR analysis, as its quality directly impacts all downstream analysis. While DEEP Picker and Voigt Fitter are increasingly used in the field, their current command-line-only interface has a learning curve, particularly for users accustomed to GUI-based applications. COLMARvista bridges this gap by offering a user-friendly interface: by the click of a button users can run DEEP Picker on the displayed spectrum, with the identified peaks automatically overlaid on the spectrum. Users also have the flexibility to manually refine the peak list, by interactively adding, moving, or deleting peaks as needed, before proceeding to the final peak fitting step by Voigt Fitter. By selecting a few parameters, such as the fitted line shape type, users can complete this task with minimal effort. Once peak fitting is complete, COLMARvista generates an updated peak list and displays the synthetically reconstructed spectrum based on the peak list and overlays it on the experimental spectrum, which allows the direct visual assessment of the deconvolution quality. All results can be downloaded for subsequent use and documentation. Alternatively, users can upload their own peak list in either NMRPipe or Sparky format for visualization and further refinement by Voigt Fitter. Figure 5 shows a selected region of a 2D ^1^H-^13^C HSQC spectrum of a biofilm metabolomics sample of Pseudomonas aeruginosa (blue) along with the reconstructed spectrum (magenta) obtained from peak deconvolution using the built-in DEEP Picker and Voigt Fitter programs. The fitted spectrum was uniformly shifted for better visualization.

**Fig. 6:**
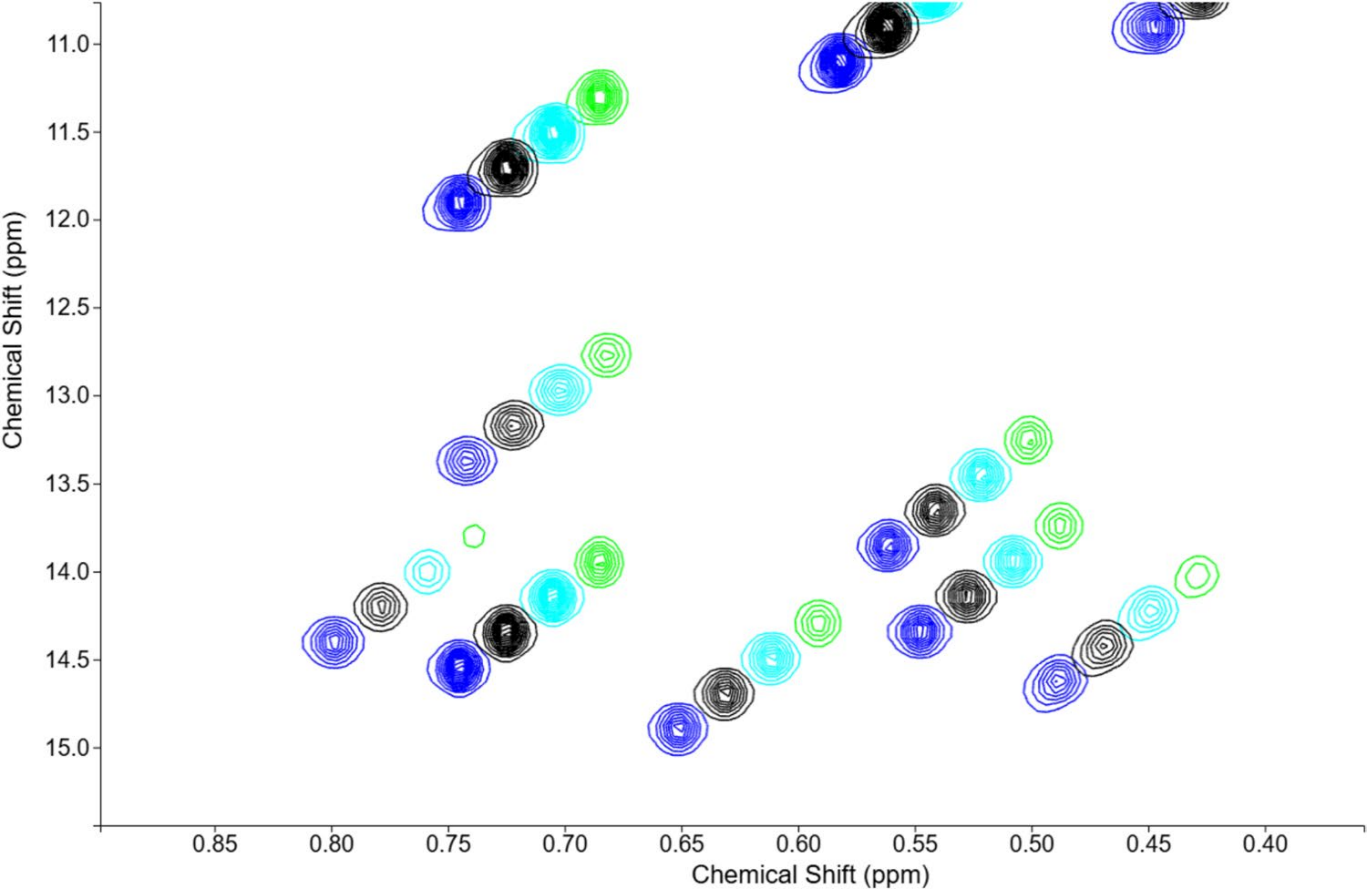
A zoomed-in region of a pseudo-3D ^15^N-*R*_1_ protein spectrum of K-Ras with four planes superimposed (blue, black, cyan, and green) corresponding to increasing relaxation delays and shifted for better visualization by COLMARvista. The contours are displayed on a linear scale to visualize relative peak volumes

COLMARvista provides an intuitive platform also for the quantitative analysis of pseudo-3D experiments. In a typical workflow, users can upload FID data and use COLMARvista’s automatic processing tool with the “Process first plane only” option selected (default). The resulting spectrum can then be visually inspected, and adjustments can be made, such as phase correction, window function, and amount of zero filling. Once satisfied, users simply uncheck the “process first plane only” option and reprocess the FID. COLMARvista will then process all planes sequentially, by applying the same optimized processing parameters, and display the contour plots of all planes overlaid for easy comparison. Next, users can run DEEP Picker and Voigt Fitter typically, but not necessarily, on the first plane to generate a deconvoluted peak list. This peak list can then serve as the starting point for the pseudo-3D peak fitting step. In this mode, Voigt Fitter ensures consistent peak positions and line shapes across all planes, allowing only peak heights to vary for each fitted peak between planes. The downloadable results are formatted similarly to those of NMRPipe, with columns labeled Z_A0, Z_A1, Z_A2, etc., to represent the relative peak heights across all planes. The relative peak heights can be directly used in downstream analysis tools to calculate parameters, such as *R*_1_ and *R*_2_ relaxation rates, or to plot profiles of CEST experiments, depending on the type of pseudo-3D data. This is illustrated in Fig. 6, where a zoomed region of a pseudo-3D protein spectrum with the superposition of four spectral planes distinguished by color (blue, black, cyan, and green) is shown after being shifted for visualization purposes.

**Fig. 7:**
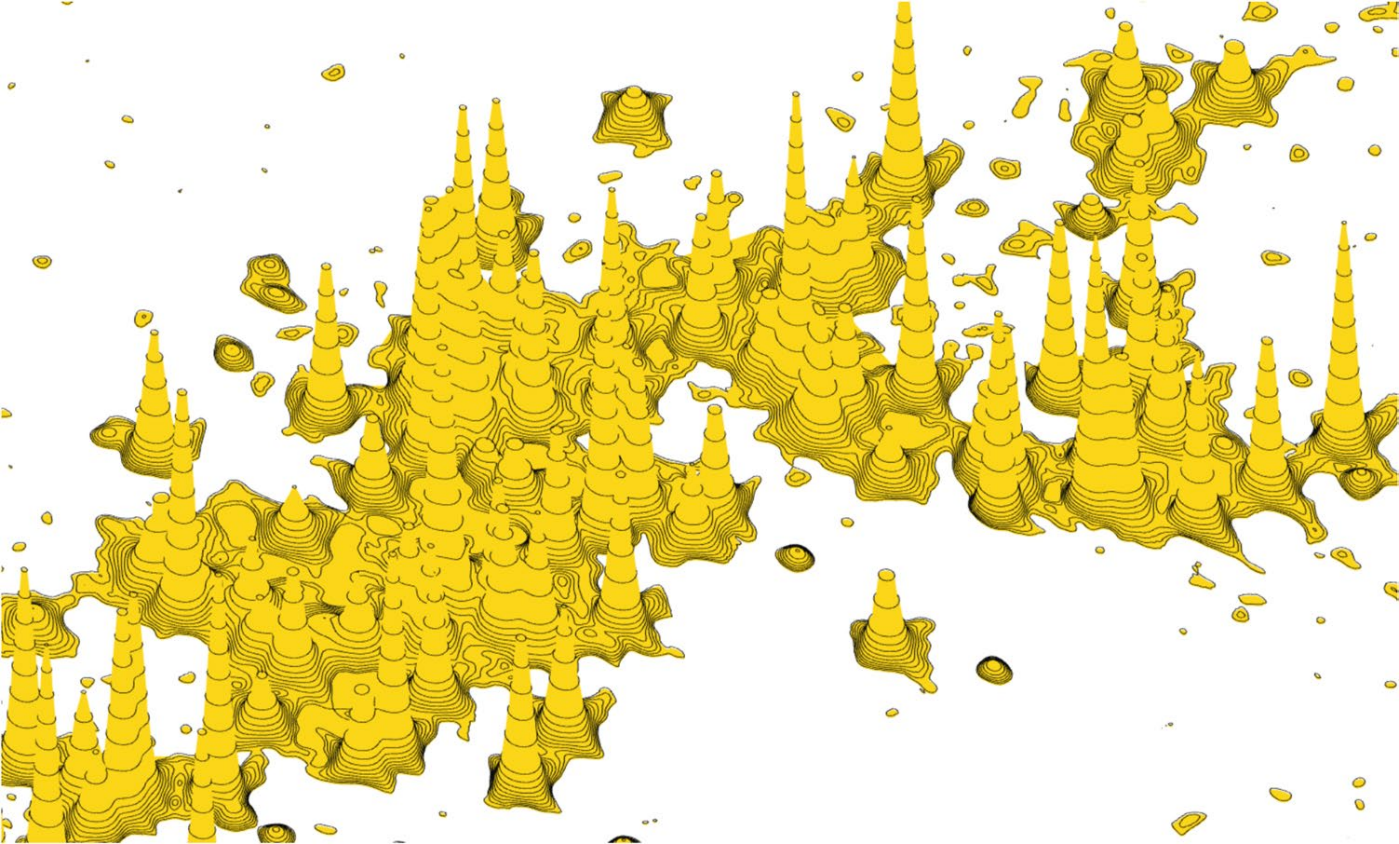
Selected region of the 3D surface plot of the ^1^H-^15^N HSQC spectrum of the IDP protein ɑ-synuclein generated by COLMARvista

As a bonus, COLMARvista can display NMR spectra also in a perspective 3D mode, either as a continuous surface (with highlighted contour lines) or as a terrace model. The 3D mode supports intuitive panning, zooming, and rotation operations using the mouse, along with optimized lighting effects. This 3D plot provides a comprehensive overview of the entire spectrum, including potential artifacts, and highlights relative peak heights in a way that aligns with human intuition. In Fig. 7a 3D surface plot of the ^1^H-^15^N HSQC spectrum of the IDP protein ɑ-synuclein is depicted. COLMARvista includes detailed instructions for all its functionalities and programming considerations, which are accessible directly within the software.

## Software access

The COLMARvista software can be downloaded directly from GitHub and run locally (https://github.com/lidawei1975/colmarvista) or accessed online as a static web server (https://lidawei1975.github.io/colmarvista). Instructions are available on the repository's Wiki page (https://github.com/lidawei1975/colmarvista/wiki). The Discussions and Pull Requests tabs of the repository can be used to report bugs, provide feedback, or collaborate. The software remains under active development, and new features will be added based on user feedback.

## Conclusion

COLMARvista was designed as a highly versatile software tool for the easy processing and visual inspection of 2D and pseudo-3D NMR data both for uniformly and non-uniformly sampled datasets fully imbedding DEEP Picker, Voigt Fitter, and SMILE. Being based on JavaScript running on all common web browsers, COLMARvista is computer-architecture and operating-system independent, which greatly facilitates its distribution, installation, use, and upkeep. Autonomous processing of spectra, including phase correction, allows users to quickly and easily display and rigorously inspect and compare their raw spectra. Integration with DEEP Picker and Voigt Fitter allows the automated deconvolution and reconstruction of the experimental spectra for the fully quantitative analysis, from compound concentrations to the extraction of site-resolved relaxation parameters. Our goal with COLMARvista is to provide the NMR community with a fast, portable and highly automated software tool for fully streamlined NMR data processing, integrated with subsequent analysis and interpretation steps. With its robust framework, COLMARvista can be easily expanded also for the handling of lower and higher dimensional spectral datasets.

## Data Availability

The code of COLMARvista is publicly available at: https://github.com/lidawei1975/colmarvista.
